# Microphthalmia‐associated transcription factor in melanoma development and MAP‐kinase pathway targeted therapy

**DOI:** 10.1111/pcmr.12370

**Published:** 2015-04-17

**Authors:** Claudia Wellbrock, Imanol Arozarena

**Affiliations:** ^1^ Manchester Cancer Research CentreWellcome Trust Centre for Cell Matrix ResearchFaculty of Life SciencesThe University of ManchesterManchesterUK

**Keywords:** melanoma, microphthalmia‐associated transcription factor, MAP kinase pathway, BRAF, MEK

## Abstract

Malignant melanoma is a neoplasm of melanocytes, and the microphthalmia‐associated transcription factor (MITF) is essential for the existence of melanocytes. MITF's relevance for this cell lineage is maintained in melanoma, where it is an important regulator of survival and balances melanoma cell proliferation with terminal differentiation (pigmentation). The *MITF* gene is amplified in ~20% of melanomas and MITF mutation can predispose to melanoma development. Furthermore, the regulation of MITF expression and function is strongly linked to the BRAF/MEK/ERK/MAP‐kinase (MAPK) pathway, which is deregulated in >90% of melanomas and central target of current therapies. MITF expression in melanoma is heterogeneous, and recent findings highlight the relevance of this heterogeneity for the response of melanoma to MAPK pathway targeting drugs, as well as for MITF's role in melanoma progression. This review aims to provide an updated overview on the regulation of MITF function and plasticity in melanoma with a focus on its link to MAPK signaling.

## Introduction

MITF is a lineage commitment factor essential for propagation of the melanocyte lineage in early development, and importantly, this role is maintained in melanoma cells. As such, MITF has gained major recognition as a central player in melanoma development and has been assigned the role of a ‘lineage survival oncogene’ (Garraway et al., 2005).

MITF is a basic helix‐loop‐helix leucine zipper (bHLH‐ZIP) transcription factor that regulates the expression from promoters containing a DNA response element that includes specific flanking nucleotides in addition to a core E‐box element usually bound by bHLH‐ZIP transcription factors (Bertolotto et al., 1996, 1998b; Lowings et al., 1992; Pogenberg et al., 2012; Yasumoto et al., 1994). For a thorough review of the specific regulation of MITF target genes, see Cheli et al. (2010).

The *Mitf* gene was originally identified by Heinz Arnheiter in 1992 as a consequence of a chance transgene insertion within the *microphthalmia* (*mi*) locus in mice. The insertion led to an albino phenotype with small eyes, hearing loss and a severe mast cell defect (Arnheiter, 2010; Hodgkinson et al., 1993). This indicated that a single gene encoded by the *mi* locus plays a crucial role in various physiological processes linked to neural crest and neuroepithelial‐derived tissues including skin pigmentation, hearing and eye development. The human homologue MITF was consequently cloned in 1994 (Tachibana et al., 1994), and soon, it became clear that the broad range of tissue‐specific functions was due to different isoforms of Mitf/MITF all transcribed from the same locus (Tachibana, 2000).

Today many alternatively spliced *MITF* transcript variants encoding different isoforms have been identified, and it is the M‐MITF isoform which is specifically expressed in the melanocyte lineage, where it regulates melanoblast propagation in early development (Opdecamp et al., 1997) as well as melanogenesis in adult melanocytes by regulating genes such as *TYROSINASE* (Yasumoto et al., 1994).

**Figure 1:**
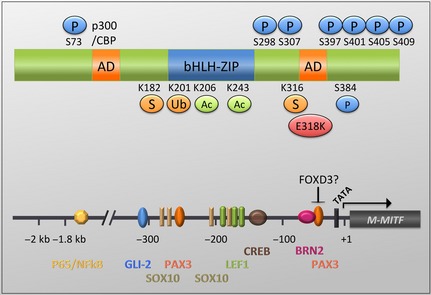
Schematic of the M‐MITF (MITF) protein and the *M‐MITF* promoter**.** Post‐translational modifications including phosphorylation (P), sumoylation (S), ubiquitination (Ub) and acetylation (Ac) are indicated as well as the E318K mutation. Transcription factors as well as their binding sites within the first 2 kb upstream of the transcription start site of the *M‐MITF* promoter are also shown.

Apart from alternative splicing, the *MITF* transcript variants are derived from different *MITF* promoters (*A, CX, MC, C, E, H, D, B, M, J*) (Levy et al., 2006; Li et al., 2010) and restricted promoter activation brings about the cell‐type‐specific expression, as it is seen in melanocytes, where the *M*‐promoter drives the expression of *M‐MITF* (Figure 1). Transcription from the different promoters leads to gene products that vary in the first exon, but are conserved in the sequences encoded by exon 2 to exon 9 of the *MITF* locus.

The various MITF isoforms display a molecular weight in the range of 50–80 kDa, whereby the M‐isoform presents as a protein with a weight between 50 and 65 kDa depending on its post‐translational modifications. Despite varying N‐termini, all isoforms contain the same functional protein domains encoded by exons 2–9 (Figure 1). This includes an N‐terminal (aa 114–132 in M‐MITF) and a C‐terminal transactivation domain (aa 324–369 in M‐MITF) (Sato et al., 1997; Steingrimsson et al., 2004; Takeda et al., 2000a). The recently resolved MITF crystal structure revealed unique features of the basic DNA binding domain (aa 208–230) and the HLH‐ZIP domain (aa 243–294) explaining both MITF's promoter specificity and its restriction to dimerize only with other MITF/TFE family members (Pogenberg et al., 2012).

M‐MITF (for simplicity called MITF in this review) is expressed in >80% of melanomas and detectable throughout all stages of melanoma development (King et al., 2001), although the conclusions regarding an overall increase or decrease in MITF expression levels during melanoma progression are controversial (Garraway et al., 2005; Salti et al., 2000; Ugurel et al., 2007). In melanoma, MITF expression is mostly heterogeneous, and immunohistological staining identifies ‘MITF‐positive’ cells expressing higher and lower levels of MITF, but also cells that lack MITF expression entirely (Konieczkowski et al., 2014; Muller et al., 2014; Sensi et al., 2011). These subpopulations of ‘MITF‐negative’ cells, which are contained within a subset of melanoma cell populations originally identified by Hoek and colleagues (Hoek et al., 2006), are characterized by the expression of the non‐canonical Wnt ligand WNT5A and the receptor tyrosine kinase AXL (Dissanayake et al., 2008; Konieczkowski et al., 2014; Muller et al., 2014; Sensi et al., 2011). Whereas in these MITF‐negative cells, factors such as WNT5A and TGF*β* appear to dominate the regulation of melanoma cell fate (Eichhoff et al., 2011; Javelaud et al., 2011), in the MITF‐positive cells, MITF is the central regulator of melanoma cell survival, proliferation and differentiation.

Because of its fundamental role in melanoma cells, the regulation of MITF's expression and function is extremely complex and dynamic. This frequently leads to contradicting findings, and this review will address some of the ‘conundrums’ in MITF regulation. Furthermore, with MITF's relevance for lineage propagation throughout the development of malignant melanoma, and the recent realization that MITF impacts on the response of melanoma to current treatments using MAPK pathway targeting drugs, this review will also address the role of MITF in melanoma development and BRAF/MEK inhibitor therapy.

## The regulation of MITF and its relevance to melanoma

The *MITF* gene is target of many regulatory mechanisms that all together orchestrate a highly dynamic control over *MITF* transcripts. In addition, post‐translational modifications add to the complexity of MITF regulation. Due to the multifactorial and dynamic nature of MITF regulation, it often varies amongst individual melanoma cells with regard to the levels of MITF expression. *In vivo,* this can partly be a consequence of different microenvironment‐dependent physiological contexts, but different genetic backgrounds of individual cells might also be involved. For instance, an *MITF* gene amplification is found in ~20% of metastatic melanomas (Garraway et al., 2005; Ugurel et al., 2007). The gene amplification in tumours can lead to an increase in copy number, but a layer of transcriptional and post‐translational regulation may ‘dampen’ the consequences of a too high gene dosage (Garraway et al., 2005). Importantly, *MITF* gene amplification is also found in patients relapsed on BRAF/MEK inhibitor therapy (Van Allen et al., 2014), suggesting that it provides a growth or survival advantage when the MAPK pathway is inhibited.

## The regulation of MITF expression at transcriptional level

Ground‐laying work by the Ballotti/Bertolotto and Fisher laboratories has identified the regulation of the mouse as well as the human M‐specific *MITF* promoter by *α*MSH‐induced cAMP signalling and the cAMP response element‐binding protein (CREB, see Figure 1) as central to the melanogenesis process (Bertolotto et al., 1998a; Busca and Ballotti, 2000; Price et al., 1998b). In melanoma cells (particularly NRAS mutant cells), *α*MSH receptor signals are often disconnected from cAMP signalling (Marquette et al., 2011), but direct activation of adenyl cyclase through agents such as forskolin or cholera toxin can initiate cAMP‐induced *MITF* transcription in the majority of melanoma cell lines, suggesting that in principle, the signalling module is preserved throughout melanoma progression.

SOX10 is a lineage‐specific transcription factor that strongly activates the *MITF* promoter (Bondurand et al., 2000; Lee et al., 2000; Potterf et al., 2000; Verastegui et al., 2000), and is crucial for MITF expression; accordingly, it is required for melanoma cell survival (Shakhova et al., 2012). SOX10 co‐operates with CRE‐binding protein (CREB) to induce MITF expression, and this interaction can bring linage specificity to cAMP signalling (Huber et al., 2003). Synergy with PAX3 and SOX10 in transactivating the *MITF* promoter is debated to occur (Bondurand et al., 2000; Potterf et al., 2000) or to be dispensable (Lee et al., 2000; Verastegui et al., 2000). A reason for these contradicting findings might be the existence of several PAX3 and SOX10 binding sites in the *MITF* promoter (Figure 1), but the different studies have not always taken all these binding sites into consideration.

The paired‐box transcription factor PAX3 is an essential regulator of the *MITF* promoter (Kubic et al., 2008; Watanabe et al., 1998), and as an inducer of MITF expression, PAX3 is required for melanocyte lineage survival early during development (Nishimura et al., 2010). Importantly, this role is maintained in melanoma, where its depletion induces apoptosis (Kubic et al., 2012; Scholl et al., 2001). In melanocyte stem cells, PAX3 – and consequently MITF – expression is suppressed by canonical TGF*β* signalling which maintains an undifferentiated state and allows for survival in the absence of PAX3 and MITF. This suppressor function of TGF*β* is maintained in adult melanocytes, where the cytokine is produced by keratinocytes and controls the level of melanogenesis (Yang et al., 2008). Importantly, TGF*β* treatment leads also to a significant reduction in PAX3 and MITF expression in melanoma cells and this correlates with the growth suppressor activity of this cytokine (Smith et al., 2013). TGF*β* can also suppress MITF expression directly through GLI2 (Pierrat et al., 2012), suggesting that reduced MITF expression is relevant for the execution of TGF*β* signalling in melanoma.

BRN2 (N‐Oct‐3) is a neuronal‐specific transcription factor that binds the *MITF* promoter in its proximal region (Figure 1). As an ERK target gene, downstream of BRAF^V600E^ BRN2 is widely expressed in melanoma cells (Goodall et al., 2004b; Wellbrock et al., 2008). Depletion of BRN2 from melanoma cells has consistently been described to reduce MITF levels (Cook et al., 2005; Thomson et al., 1995; Thurber et al., 2011; Wellbrock et al., 2008), indicating that BRN2 is required to drive MITF expression. In line with this, BRN2 increases transcription from the *MITF* promoter in a panel of BRAF mutant melanoma cells (Wellbrock et al., 2008). However, in some cells, MITF expression is unaltered after BRN2 depletion, and in one cell line, MITF protein levels are even increased (Goodall et al., 2008; Thurber et al., 2011). In line with this, in some cells, BRN2 suppresses *MITF* promoter activity (Goodall et al., 2008). These opposing observations might be due to different signalling backgrounds; for instance, the transcriptional activation of the *MITF* promoter happens downstream of BRAF^V600E^ signalling (Wellbrock et al., 2008) and the suppressor function occurs in *BRAF* ^*WT*^ B16 cells (Goodall et al., 2008), where BRAF signalling does not regulate BRN2. A similar situation is found in 501mel cells, where hyperactive *β‐catenin* ^*D32H*^ regulates BRN2 expression (Goodall et al., 2004a, 2008). In these cells, *β*‐catenin^D32H^ also constitutively drives *MITF* expression (Arozarena et al., 2011a; Widlund et al., 2002). This appears to create a *β*‐catenin/BRN2/MITF network, in which BRN2 can act as a suppressor but the role of BRAF^V600E^ signalling to BRN2 or MITF is unclear. In summary, in order to fully dissect the regulation of MITF by BRN2, future analyses need to consider the genetic and signalling background of melanoma cells.

Wnt3 regulates MITF expression through *β*‐catenin, which binds the *MITF* promoter through a TCF1 binding site at ‐199/‐193 (Takeda et al., 2000b) (Figure 1) and in zebrafish induces *mitf*‐driven differentiation from the neural crest during early development (Dorsky et al., 2000). Wnt3 also regulates differentiation in B16 melanoma cells and reduces the proliferation of human melanoma cells (Chien et al., 2009). On the other hand, ectopic overexpression of *β*‐catenin stimulates melanoma cell growth in a MITF‐dependent manner (Widlund et al., 2002), suggesting an altered transcriptional activity of *β*‐catenin in the absence of a Wnt3 signalling context.

Mice transgenic for mutant ‘oncogenic’ *β*‐catenin do not develop melanoma, but display reduced melanoblast migration visible as white belly (Delmas et al., 2007; Gallagher et al., 2013). In line with this observation, mutant *β*‐catenin‐expressing cells (e.g. 501mel) display no invasive activity *in vitro* and *in vivo* (Arozarena et al., 2011a; Carreira et al., 2006; Chapman et al., 2014), and this low invasive activity is linked to MITF expression, which is induced by *β*‐catenin in these cells. Mechanistically, MITF can interfere with *β*‐catenin target gene specificity (Schepsky et al., 2006), and in 501mel cells, this leads to reduced *β*‐catenin‐mediated expression of *MT1‐MMP*, a protease essential for invasion (Arozarena et al., 2011a). Importantly, when mutant *β*‐catenin is expressed in Braf^V600E^/Pten^‐/‐^‐ or NRas‐driven melanoma in mice, this significantly enhances the development of metastasis (Damsky et al., 2011; Gallagher et al., 2013). This suggests strikingly opposing functions of *β*‐catenin in early and late stages of melanoma development, but so far, the role of MITF downstream of mutant *β*‐catenin particularly in the stages of metastasis is unknown.

Regulation by TNF‐*α* can induce MITF expression in melanoma cells through IKK/NFkB signalling. Thereby, p65/NFkB binds to a distal region in the MITF promoter (‐1870/‐1879) and stimulates transcription (Smith et al., 2014). This regulation has gained relevance in the context of microenvironment signalling from immune cells, where macrophage‐derived TNF‐*α* appears to be required for the maintenance of MITF expression during melanoma growth (Smith et al., 2014). Intriguingly, in some cells, TNF‐*α* suppresses MITF expression, and an ‘activated NFkB signalling’ signature correlates with highly reduced MITF expression (Konieczkowski et al., 2014; Landsberg et al., 2012). Notably, this signature strictly correlates with the expression of the receptor tyrosine kinase AXL and thus may exist predominantly in the previously mentioned ‘MITF‐negative’ cells (Sensi et al., 2011). However, how AXL or the related gene signature is linked to NFkB suppressing MITF expression rather than inducing it remains to be clarified.

A couple of other transcriptional regulators of MITF expression have been identified, where follow‐up studies in human melanoma are missing. One such factor is FOXD3, which has been identified as a suppressor of *mitf* expression in zebrafish and quail (Thomas and Erickson, 2008), where it is a crucial factor in lineage commitment from the neural crest. Initially, a FOXD3 binding site was mapped to the region of the proximal PAX3 binding site (Figure 1), and subsequently, it was shown that FOXD3 interacts with PAX3, thereby preventing it from activating the *mitf* promoter (Thomas and Erickson, 2009). In this context, it is intriguing that in melanoma cells, BRAF suppresses FOXD3 (Basile et al., 2012), which would allow MITF expression to prevail. However, due to the lethality of depleting FOXD3 from the NC lineage in mice (Hanna et al., 2002), little is known about the FOXD3 regulation of MITF in mammalian cells.

Finally, MITF expression is also regulated through microRNAs (miRs) (Bell and Levy, 2011). Amongst them are miR‐137 (Bemis et al., 2008), miR‐148 (Haflidadottir et al., 2010) and miR‐182 (Segura et al., 2009). However, the consequence of MITF regulation by miRNAs is not always clear most probably due to the fact that these miRNAs target a wide range of other mRNAs. A more complex regulation of MITF expression is thought to occur via miR‐211, which targets the MITF transcriptional regulator BRN2 (Boyle et al., 2011) Although the actual effect of miR‐211 on MITF expression was not shown by Boyle et al. and still has to be demonstrated, the fact that miR‐211 expression is itself regulated by MITF (Levy et al., 2010; Mazar et al., 2010) suggests a potential feedback loop in which MITF expression levels are balanced through miR‐211 and BRN2 expression.

## The regulation of MITF at post‐translational level

MITF is target to numerous post‐translational modifications, amongst them are sumoylation**,** ubiquitination and acetylation**,** modifications that contribute to the regulation of MITF's turnover and transcriptional activity.

Sumoylation at K182 and K316 (Figure 1) has been described to reduce the synergistic transcriptional activity of MITF at the *DCT* and *TRPM1* promoter, respectively (Miller et al., 2005; Murakami and Arnheiter, 2005). While both studies observe no impact of sumoylation on MITF's protein stability, nuclear localization or DNA binding efficiency, Miller et al. identify PIAS3 as a promoter of sumoylation, whereas Murakami and Arnheiter exclude any involvement of PIAS proteins in the process. The difference might be due to the different cell systems used (HEK293, COS‐7), neither of which are melanocytic cell lines.

The sumoylation of MITF gained major relevance, when it was discovered that the recurrent germ line mutation E318K, which interferes with MITF sumoylation at K316, predisposes to melanoma and renal carcinoma and links MITF to familial melanoma (Bertolotto et al., 2011; Yokoyama et al., 2011). At the functional level, the E318K mutation increases the binding of MITF to a subset of target genes and thus results in differential transcriptional regulation. Of note, in osteoclasts, E316 sumoylation regulates MITF's interaction with the co‐activator FUS and the chromatin remodelling ATPase BRG1 (Bronisz et al., 2014), and because the BRG1 containing SWI/SNF complex is regulating MITF‐mediated transcription from, for example, the *TRP1* and *TYROSINASE* promoter (De La Serna et al., 2006), the E318K mutation might have a direct impact on MITF's transcriptional activity. Nevertheless, while a phenotypic characterization of nevi and tumour patterns has been described recently in a small group of MITF E318K melanoma patients (Sturm et al., 2014), a detailed functional analysis explaining why this unique mutation has such a major impact on melanoma development is lacking so far.

MITF can be ubiquitinated at K201 (Figure 1), and mutating K201 as well as inhibiting the proteasome results in stabilization and accumulation of the MITF protein (Wu et al., 2000; Xu et al., 2000), indicating that ubiquitination triggers MITF degradation. Indeed, when protein synthesis is inhibited using cycloheximide, the basal half‐life of MITF is 30 min in HEK293T cells (Zhao et al., 2011) up to 2.5 h in non‐stimulated melanocytes (unpublished observation). On the other hand, overexpression of the deubiquitinase USP13, which has been identified as a key regulator of MITF turnover, can enhance the basal half‐life of the MITF protein to up to 4 h (Zhao et al., 2011). This suggests a rapid basal turnover of MITF that is tightly controlled, as it is found with many central transcriptional regulators.

Apart from sumoylation and ubiquitination, other lysine residues in MITF appear to be the target of acetylation, and K206 and K243 have been mentioned to be acetylated (Cheli et al., 2010). Acetylation, similar to sumoylation, could control MITF's target gene specificity and as such might be involved in directing the MITF target gene repertoire. Acetylation and also ubiquitination have been linked to ERK/MAPK signalling (Price et al., 1998a; Wu et al., 2000; Xu et al., 2000), which provides a direct link of MITF abundance and function with the most predominantly deregulated pathway in melanoma.

Despite its relevance for melanoma, the understanding of MITF as a target of phosphorylation is relatively limited and sometimes ambiguous. The only comprehensively studied phosphorylation event was initially identified by the Fisher laboratory in 1998 (Hemesath et al., 1998). The phosphorylation occurs at S73 and is performed by ERK (Figure 1). Since then, other studies have confirmed that ERK phosphorylates MITF *in vitro* and in cells (using phospho‐specific antibodies) and that ERK2 directly binds the N‐terminus of MITF (Molina et al., 2005; Sato‐Jin et al., 2008).

Apart from ERK itself, the ERK target p90RSK1 has been shown to phosphorylate S409 downstream of activated KIT (Wu et al., 2000). Purified RSK1 is able to phosphorylate a 30aa S409 containing MITF peptide *in vitro,* and RSK1 can be co‐precipitated with MITF from melanoma cells after stimulation with KIT ligand, but not from untreated cells (Wu et al., 2000). Intriguingly, although this demonstrates a role for RSK1 in MITF phosphorylation, so far no follow‐up study has studied the action of p90RSK1 in the phosphorylation of MITF at S409 by, *for example,* using RSK1‐specific siRNAs or defining the binding site, and it might well be possible that other kinase also contributes to S409 phosphorylation when KIT is not involved.

S298 gained attention**,** because its mutation to a proline had been linked to Waardenburg syndrome II (Tassabehji et al., 1995). S298 can be phosphorylated by GSK3 *in vitro*, and mutating S298 abolished MITF's *in vitro* DNA binding ability and severely affected its transcriptional activity (Takeda et al., 2000a). However, a recent study did not observe these effects, and the analysis of available sequence databases suggests that the S298P mutation is a rare polymorphism functionally equivalent to the wild type (Grill 2013). While this questions the role of S298 as GSK3 phosphorylation site, recently three novel GSK3 sites S397, S401 and S405 were identified (Figure 1), and mutation of these sites or inhibition of GSK3 enhanced MITF's stability (Ploper et al., 2015).

S307 and S384 were found by phosphoproteomics (Cantin et al., 2008; Olsen et al., 2010). While no information is available regarding S384**,** S307 acts as p38 phosphorylation site downstream of RANKL in osteoclasts (Mansky et al., 2002) and is involved in the interaction of MITF with FUS and BRG1 (Bronisz et al., 2014), but strikingly nothing is known in this context in melanocytic cells.

## The regulation of MITF by the ERK/MAPK pathway

**Figure 2:**
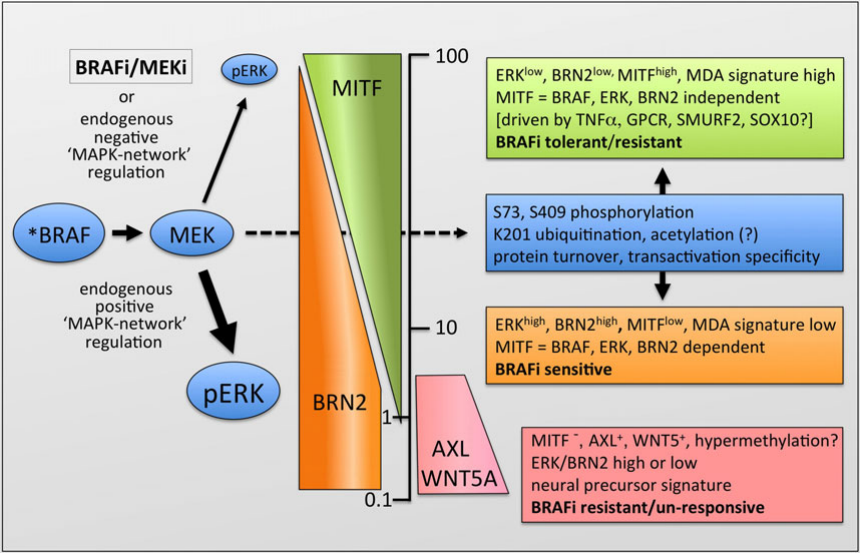
Model of MITF regulation through ERK downstream of oncogenic BRAF. Three types of melanoma cell populations can be classified by their MITF, AXL and WNT5A status. In MITF‐positive/AXL ^‐^/WNT5A^‐^ melanoma cells, MITF expression is regulated through the ERK target BRN2. When ERK activity is high, the BRAF/BRN2 contribution to transcription from the *M‐MITF* promoter is high. High ERK activity also leads to an increased MITF protein turnover; as a consequence, MITF protein levels are low. The opposite applies to low ERK activity. BRAF also contributes to the post‐translational regulation of MITF via MEK and ERK, and this will impact on MITF function at any level of MITF expression. In MITF‐negative/AXL ^+^/WNT5A^+^ cells, ERK activity as well as BRN2 expression can be high or low and are uncoupled from MITF expression.

As already mentioned, MITF is the target of the ERK/MAPK pathway at various levels including its transcription and its protein turnover and function. This possibly reflects a general concept of a cell‐type‐specific central transcriptional regulator being controlled by a signalling pathway fundamental to the biology of this cell type. Importantly, this link appears to be maintained in melanoma where oncogenic BRAF or NRAS hyperactivates the MAPK pathway. Indeed, MITF is essential for the maintenance of oncogenic BRAF‐driven melanoma *in vivo* (Lister et al., 2014), and the level of MAPK pathway activation appears to be critical for MITF abundance and function in melanoma cells (Figure 2).

## The ERK/MAPK pathway and MITF function

The relevance of the MAPK pathway for MITF was first highlighted by the discovery that it is phosphorylated by ERK downstream of the receptor tyrosine kinase KIT (Hemesath et al., 1998). The ERK‐mediated phosphorylation of MITF at S73 enhances its transcriptional activity at the *TYROSINSE* promoter in the presence of constitutively active RAF kinase (Hemesath et al., 1998) and a S73A mutation impaired MITF's transcriptional activity (Price et al., 1998a; Wu et al., 2000). Strikingly, despite the deregulation of RAF kinase signalling in melanoma and its relevance for melanoma development, so far it is not known what the global consequence of ERK‐mediated phosphorylation for MITF's transcriptional activity is. What has been shown is that S73 phosphorylation is required for its interaction with the acetyltransferase p300 in human melanoma cells carrying a BRAF mutation (Price et al., 1998a), an observation that suggests a role of MAPK signalling in MITF acetylation (Figure 2). However, in melanocytes or BRAF^WT^ B16 melanoma cells, MITF co‐immunoprecipitates with p300 in a S73 phosphorylation‐independent manner (Sato et al., 1997), possibly because in these cells, the MAPK pathway is not significantly activated and the interaction is regulated in a different manner. S409 phosphorylation has also been implicated in the regulation of MITF's transcriptional activity; however, this appears to be closely linked to S73 phosphorylation (Wu et al., 2000). A S409A mutation on its own had no major effect on MITF's ability to transactivate the *Tyrosinase* promoter, but a MITF S73A/S409A mutant appeared to actually suppress basal transcription from the promoter (Wu et al., 2000). This is a striking observation, considering that phosphorylation of S409 was shown to play a role in the interaction of MITF with PIAS3 and its ability to reduce MITF's transcriptional activity, because a S409D mutant cannot interact with PIAS3 and suppress promoter transactivation (Levy et al., 2003).

Despite the compelling evidence of the relevance for S73 and S409 phosphorylation for MITF function, a recent *in vivo* study performed by the Steingrimsson laboratory and based on a BAC transgene rescue has challenged these *in vitro* data (Bauer et al., 2009). Re‐expression of various MITF S73/S409 variants from BACs in MITF‐deficient mice demonstrated that mutation of either S73 or S409 to an alanine does not significantly impair MITF function with regard to its regulation of pigmentation and eye development during development. While this finding is conceivable regarding the rather ‘mild’ *in vitro* effects of these single mutants, the observation that a S73A/S409A double mutant was also able to rescue the pigmentation and eye phenotype is puzzling, considering the dramatic effect on MITF function observed *in vitro* (Wu et al., 2000). An *in vivo* rescue was also seen with exon 1/2 deletion mutants (Bauer et al., 2009), which is in line with previous findings where an exon 2B loss does not produce an obvious phenotype (Bismuth et al., 2008).

It might be that redundancy and further modifications of MITF in a complex heterogeneous *in vivo* situation, not observed in isolated cell cultures, can explain the discrepancy observed with regard to MITF's role in development and melanocyte differentiation. However, as S73/S409 are target sites for ERK‐/RSK1‐mediated phosphorylation, assessing the BAC transgenic mice in the context of deregulated MAPK signalling in an NRAS and/or BRAF mutant background will be of major importance and could reveal essential regulatory mechanisms relevant for melanoma.

## The ERK/MAPK pathway and MITF degradation

Further evidence for a crucial regulation of MITF by the MAPK pathway came from the observation that MEK inhibition can slow down the MITF turnover that is observed in the presence of cycloheximide (Wu et al., 2000), thus implicating MEK/ERK signalling in MITF degradation. Strikingly however, cAMP signalling activates the MAPK pathway in melanocytes and melanoma cells (Busca et al., 2000), but no degradation is observed under these conditions (Price et al., 1998b). These apparently contradicting findings highlight a critical aspect of MAPK signalling that must be considered when trying to understand its function in melanoma. The MAPK signalling network is highly dynamic with many inbuilt ‘positive’ or ‘negative’ regulatory feedback mechanisms, and this can result in either a strong (or sustained) or weak (or transient) activation of ERK (Marshall, 1995; Von Kriegsheim et al., 2009; Wellbrock et al., 2004a) (Figure 2).

Indeed, due to a MKP1‐/DUSP1‐mediated feedback regulation, ERK is only transiently activated during early stages of cAMP signalling in melanocytes (Wellbrock et al., 2002). This ensures that MITF is protected against ERK‐triggered degradation in later phases of cAMP signalling, when CREB‐induced MITF transcription has ceased, yet protein levels are maintained at a high level to induce differentiation (Price et al., 1998b; Wellbrock et al., 2002). In contrast, mitogenic signals that induce a strong and sustained activation of ERK lead to reduced MITF protein levels (Wellbrock and Marais, 2005; Wellbrock et al., 2002). In melanocytes, strong activation of ERK is most probably achieved by the synergistic action of growth factors (Bohm et al., 1995), which explains the requirement of a cocktail of growth factors to stimulate melanocyte proliferation *in vitro*. Similarly, a BRAF V600E mutation hyperactivates ERK and stimulates proliferation in melanocytes (Wellbrock et al., 2004b). Moreover, ectopic expression of BRAF^V600E^ in human melanocytes reduces MITF protein levels (Wellbrock and Marais, 2005). Although it is not entirely clear whether this downregulation is required for proliferation or just a consequence of ERK activation, forced overexpression of MITF in murine BRAF‐transformed melanocytes inhibits proliferation (Wellbrock and Marais, 2005), suggesting that the control of MITF expression levels by the MAPK pathway is important in BRAF‐driven melanoma growth.

While it is well established that MAPK signalling is involved in MITF turnover (Wellbrock and Marais, 2005; Wellbrock et al., 2002; Wu et al., 2000), there is controversy at the mechanistic level. Wu et al. showed that both phosphorylations of S73 and S409 are required for efficient proteasome‐mediated degradation, which is in line with the finding that mutation of S73 alone does not stabilize the protein in oncogenic BRAF‐transformed melanocytes (Wellbrock and Marais, 2005). However, Xu et al. found that the mutation of S73 to alanine abolished MITF ubiquitination in a cell‐free extract (Xu et al., 2000), suggesting that S73 is the critical residue for this biochemical modification. What really ‘muddies the water’ is the fact that numerous studies assign ‘ERK‐mediated degradation’ as an explanation for their observations, but a closer look reveals that the MITF protein ‘disappears’ at for instance 4–6 h after the particular treatment, while ERK phosphorylation/activity and the MITF shift (turning MITF into the degradable form) are rather transient and only occur within the first 0.5–1 h. Also, many studies that use cycloheximide to study MITF degradation as the consequence of MAPK pathway activation do not perform a control assessing the basal turnover of MITF in an unstimulated situation. As mentioned before, the basal half‐life of MITF in the absence of ERK activation can range between 30 min and 2.5 h (Zhao et al., 2011) and so far it has not been thoroughly investigated how much ERK‐mediated phosphorylation enhances this turnover. Thus, although strong constitutive activation of the MAPK pathway clearly promotes MITF degradation, the situation appears to be more complex. The recent discovery of GSK3 as regulator of MITF stability might help to shed more light on this, because S409 appears to act as essential priming site for GSK3‐mediated phosphorylation (Ploper et al., 2015). This suggests a possible novel link between MAPK and Wnt signalling in the context of MITF regulation.

## The regulation of MITF transcription by oncogenic BRAF

The role of the MAPK pathway in MITF's transcriptional regulation is controversial, and studies using MEK or BRAF inhibitors report highly contradictory effects involving either reduction or upregulation of MITF‐mRNA levels (Diwakar et al., 2008; Haq et al., 2013a,b; Johannessen et al., 2013; Kono et al., 2006; Wellbrock et al., 2008). Oncogenic BRAF uses BRN2 as a crucial link to the *MITF* promoter by stimulating BRN2 expression (Wellbrock et al., 2008). This allows BRAF to assume control over the regulation of MITF expression, and BRN2 depletion abolishes the ability of BRAF^V600E^ to activate the *MITF* promoter in human melanoma cells (Wellbrock et al., 2008). Of note, in murine BRAF^WT^ cells (B16, melan‐a), ectopic expression of either BRAF^V600E^ or BRN2 suppresses the *MITF* promoter (Goodall et al., 2008; Wellbrock and Marais, 2005; Wellbrock et al., 2008). However, it is not clear whether this is a difference between mouse and human cells or the fact that these cells normally do not use BRAF/BRN2 signalling to regulate MITF.

In distinct areas of melanoma biopsies, BRN2 and MITF expression levels are found inversely correlated (Goodall et al., 2008; Thurber et al., 2011). Although this has been interpreted as a reflection of a BRN2 suppressor role, the fact that, in other areas of the same tumour, BRN2 and MITF are intensely co‐expressed (Thurber et al., 2011) suggests that the situation is more complex and that local microenvironment‐derived signals are also relevant. Indeed, when melanoma spheres are exposed to embryonic fibroblast‐conditioned medium, the otherwise predominant co‐expression of BRN2 and MITF switches to an inversely correlated expression (Thurber et al., 2011). In this context, it is noteworthy that microenvironment‐derived signals can locally increase MAPK signalling, which will enhance BRN2 expression. Coincidently, strong activation of MAPK signalling will reduce MITF protein levels through degradation, and this could explain why high BRN2 expression correlates with low MITF expression in areas where the pathway is hyperactivated by local signals. Indeed, phospho‐ERK signals display significant heterogeneity within tumours (Houben et al., 2008), and considering phospho‐ERK levels when assessing BRN2 and MITF expression levels could help to shed more light on their regulation in melanoma.

Overall, in BRAF mutant/MITF‐positive melanoma cells, a BRAF/BRN2 rheostat appears to control MITF basal expression levels (Figure 2). When mutant BRAF stimulates strong MAPK pathway activation, BRN2 is expressed at high levels and required for BRAF controlled MITF expression (Wellbrock et al., 2008) – no suppressor role for BRN2 in this context has been shown so far. It seems essential that BRAF maintains MITF at a certain expression level to ensure efficient survival and proliferation, but prevents MITF from triggering a cell cycle exit and differentiation (Gray‐Schopfer et al., 2007; Wellbrock et al., 2008). The relevance of levels of functional MITF protein for BRAF^V600E^ has been elegantly shown by the Patton laboratory using temperature‐sensitive zebrafish mutants, in which alternative splicing creates a functional inactive MITF that can act in a dominant‐negative fashion on any correctly spliced functional MITF (Zeng et al., 2015). Thus, the levels of functional MITF protein are lower, which is reflected in a delay in differentiation in mutant embryos, suggesting that melanocytes spend more time in an un‐differentiated/proliferative state (Zeng et al., 2015). This might be crucial, because expression of BRAF^V600E^, which in WT zebrafish triggers nevi formation (Patton et al., 2005), induces melanoma in these mutants (Lister et al., 2014). This not only emphasizes the relevance of the BRAF‐MITF link, but also confirms that BRAF^V600E^ co‐operates with appropriate MITF levels in driving melanoma initiation.

When BRAF, MEK or ERK are either less active or inhibited BRN2 levels drop, and to maintain survival, other factors must regulate MITF expression in a BRAF‐independent manner (this would apply for BRN2 either being an activator or suppressor). It appears that these other factors are stronger activators of the *MITF* promoter, as for instance in a panel of 88 short‐term melanoma cultures, high MITF RNA expression was found in a ‘low MAPK gene set’, but MITF expression was low in a ‘high MAPK gene set’ (Haq et al., 2013a). As such, MITF expression would be higher when ERK activation is low (Figure 2), which would be further manifested by greater protein stability.

The BRAF/BRN2 rheostat model suggests that in ERK^high^/BRN2^high^ cells, the MITF‐driven survival and proliferation signals are very much controlled by and, as such, dependent on an activated ERK/MAPK pathway downstream of BRAF. ERK^high^/BRN2^high^ cells would therefore be much more sensitive to BRAF inhibition than cells, where the pathway is less active and contributes less to MITF expression. As such, in ERK^low^/BRN2^low^ cells, MITF‐mediated proliferation and survival signals are maintained through other BRAF‐independent signalling pathways, and cells are more tolerant to MAPK pathway inhibition (Figure 2).

In this model, expressions of AXL and WNT5A define a distinct MITF‐negative population of melanoma cells, where MITF expression is not linked to BRN2 and the MAPK pathway and cells are MAPK pathway inhibitor unresponsive (Figure 2). In line with this, there is no correlation between MITF and BRN2 expression in AXL^+^/WNT5A^+^ melanoma cells (unpublished data). It is not clear what upregulates AXL or WNT5A expression, but BRAF inhibition in a panel of BRAF^mut^/AXL^+^ melanoma cells does not reduce AXL expression (Konieczkowski et al., 2014). Furthermore, there is no correlation of AXL expression with BRAF mutation status, but rather an overrepresentation in NRAS mutant melanoma ((Sensi et al., 2011) unpublished data). AXL also does not appear to induce the low MITF expression, because AXL knock‐down does not lead to re‐expression of MITF (Sensi et al., 2011). However, WNT5A efficiently suppresses MITF (Dissanayake et al., 2008). Thus, AXL^+^/WNT5A^+^ cells are governed by a distinct signalling network, in which mutant BRAF appears not to play a major role. Indeed, AXL^+^/WNT5A^+^ melanoma cells are resistant to MAPK pathway inhibitors (Konieczkowski et al., 2014; Muller et al., 2014) and this might, at least in part, be due to the fact that the regulation of MITF expression is disconnected from BRAF.

## The role of MITF in MAPK pathway targeted therapy

The use of BRAF and MEK inhibitors in stratified patients has led to significant successes in the targeted therapy of melanoma, but the development of resistance is creating a major challenge (Salama and Flaherty, 2013; Wellbrock, 2014). Growing evidence points to MITF as marker for innate and acquired resistance to MAPK pathway inhibitors. However, the situation is complex and strikingly both enhanced MITF expression and lack of MITF expression have been linked to resistance to BRAF and MEK inhibitors.

Several studies have shown that high MITF expression can overcome the cytotoxic effects of BRAF and MEK inhibitors. In MEK inhibitor‐resistant melanoma cells, MITF expression can be highly upregulated as a consequence of deregulated TGF*β* signalling (Smith et al., 2013). Thereby, cells that overexpress the SMAD‐E3 ligase SMURF2 display increased PAX3 expression, which in turn induces MITF expression. On the other hand, treatment of non‐resistant melanoma cells with TGF*β* suppresses PAX3 and MITF expression and sensitizes melanoma cells to MEK inhibition (Smith et al., 2013). This effect is also seen in patients, where increased expression of TGF*β* during treatment correlates with a better response (unpublished data).

The protective effect produced by MITF upregulation is probably due to the fact that MITF regulates multiple survival and anti‐apoptotic genes. In line with this, the MITF target *BCL2A1* can antagonize BRAF and MEK inhibition (Haq et al., 2013b). Moreover, MITF contributes to BRAF inhibitor resistance through the regulation of *BCL2A1* expression, particularly when the *BCL2A1* gene is amplified (Figure 2). MITF can also confer resistance through the regulation of its target gene PGC1*α*, which enhances oxidative phosphorylation (Haq et al., 2013a). Indeed, increased MITF and PGC1*α* expression is found in patients with acquired resistance, but mTORC1/2 inhibition can overcome PGC1*α* upregulation in melanoma cells, rendering them sensitive to MEK inhibition (Gopal et al., 2014).

Not surprisingly, MITF expression is also regulated through the microenvironment, and macrophages can induce MITF expression in melanoma cells through secreted TNF‐*α* (Smith et al., 2014). Moreover, this can create a situation of enhanced tolerance during BRAF and MEK inhibitor therapy, because the number of tumour‐associated macrophages increases in response to the inhibitors, and this correlates with increased expression of MITF during treatment (Smith et al., 2014). Finally, G‐protein‐coupled receptor/cAMP‐induced MITF upregulation confers MAPK pathway inhibitor resistance, and enhanced CREB phosphorylation is detected in tumours of relapsed patients (Johannessen et al., 2013).

Strikingly, two other crucial MITF regulators FOXD3 and SOX10 are also found upregulated in response to MAPK pathway inhibition (Abel et al., 2013; Sun et al., 2014). Although they are contributing to inhibitor resistance through MITF‐independent mechanisms and MITF expression has not been assessed in these studies, it suggests that the long‐term inhibition of MAPK signalling *in vivo* majorly impacts on the MITF regulatory network. While this further emphasizes the close connection of the MAPK pathway with the regulation of MITF in melanoma cells, the role of SOX10 and FOXD3 in regulating MITF downstream of ERK under physiological conditions in melanocytes is so far unknown.

Overall, these recent findings suggest that blocking MITF‐induced survival signalling would enhance the response to MAPK pathway inhibitors, and indeed targeting, for instance, the macrophage‐TNF‐*α*‐mediated MITF upregulation using IKK inhibitors profoundly sensitizes to BRAF and MEK inhibitors (Smith et al., 2014). Alternatively, inhibiting MITF ‘downstream activities’ by targeting MITF survival target genes using obatoclax can sensitize to vemurafenib (Haq et al., 2013b).

However, even though there being an argument for suppressing MITF expression or function during MAPK pathway inhibitor treatment, there might be a flip side to such an approach; in line with the upregulation of MITF in response to MAPK pathway inhibition, a significant upregulation in melanoma differentiation antigen (MDA) expression (i.e. MITF target genes such as *TYRP1*,* MART‐1/MLANA* or *DCT/TYRP2*) is detectable in tumours of patients on treatment (Frederick et al., 2013). Most importantly, this upregulation correlates with an increased CD8+ T lymphocyte infiltration (Frederick et al., 2013), a situation favourable in the context of immunotherapy. Thus, enhanced MITF expression during treatment could be advantageous for adoptive immunotherapy approaches, and its suppression would be counterintuitive.

A role for MITF upregulation in increased tolerance and resistance to MAPK pathway inhibitors is further supported by the fact that some patients relapse with a *MITF* gene amplification (Van Allen et al., 2014). Remarkably however, the absence of MITF marks a AXL^+^/WNT5A^+^ population of melanoma cells – previously identified as the ‘Hoek's invasive signature cohort’ (Hoek et al., 2006) – that is unresponsive to MAPK pathway inhibitors (Anastas et al., 2014; Konieczkowski et al., 2014; Muller et al., 2014; O'connell et al., 2013). While MITF expression does not seem to be relevant in this melanoma cell population, both WNT5A and AXL expressions correlate with clinical response and therapy resistance, suggesting that assessing the AXL/WNT5A expression status could be used as prognostic and/or predictive marker.

## The function of MITF in melanoma development

The function of MITF in melanoma development is complex, which is partly due to its dynamic regulation, for example through the genetically activated ERK/MAPK pathway in addition to microenvironment‐derived signalling, but also due to its broad range of target genes. As a ‘master regulator’ of melanoma cell biology, MITF's most important role is probably its function in proliferation and survival, which also explains why MITF expression is maintained throughout tumour progression. In this context, MITF regulates oxidative metabolism and allows melanoma cells to adapt to local nutrient conditions (Haq et al., 2013a). On the other hand, MITF contributes to differentiation, which involves exiting the cell cycle and triggering the melanogenesis programme, and this function appears to frequently sustain throughout melanoma development considering the often highly pigmented lesions observed even in late stage melanoma.

## MITF is required for cell cycle progression and survival

**Figure 3:**
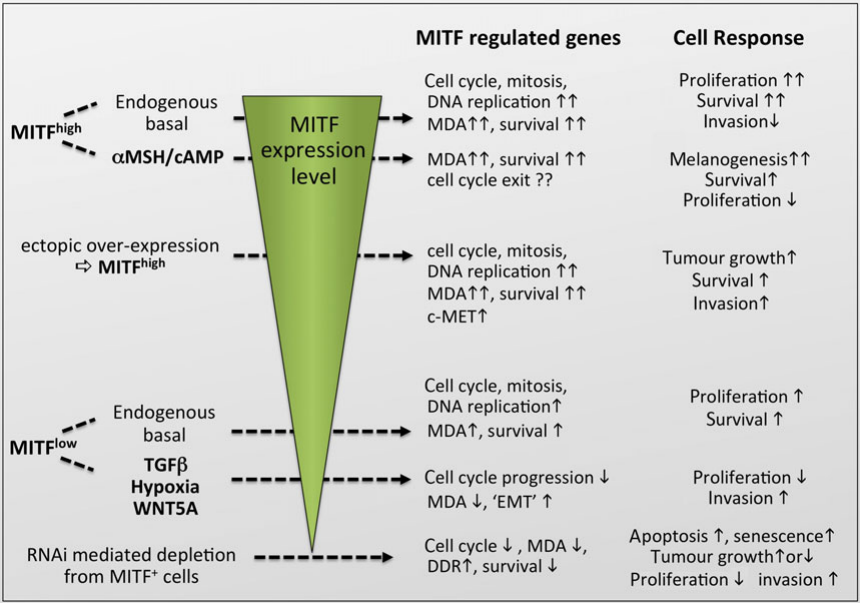
Individual functions of MITF at different expression levels**.** High MITF expression can be endogenous or the result of cAMP‐induced upregulation. MITF expression can be intrinsically low or induced through exogenous factors such as TGF *β*, WNT5A or hypoxia. Concomitant signalling will impact on MITF's function with regard to target gene recognition and transactivation from the respective promoters, and ultimately on the cellular response. At all levels, MITF regulates cell cycle progression, but at low levels, its contribution is less.

Depletion of MITF from high or low MITF‐expressing cells results in a G1 arrest (Carreira et al., 2006; Wellbrock et al., 2008), demonstrating that it is essential for driving melanoma cell proliferation independently of its expression level (Figure 3). MITF can promote cell cycle progression by inducing the expression of *CDK2* and *CDK4* (Du et al., 2004; Wellbrock et al., 2008). Furthermore, MITF suppresses P27KIP1, but the regulation is more complex and occurs at the post‐translational level through SKIP2‐mediated degradation (Carreira et a l., 2006). MITF also regulates genes involved in DNA replication and mitosis (e.g. *BRCA1*,* TERT*,* CCNB1*,* AURKB*) (Beuret et al., 2011; Strub et al., 2011). Hence, depletion of MITF can result in DNA damage and mitotic defects, and depending on the cellular background of an individual cell, MITF depletion will induce either senescence or apoptosis in response to these insults (Giuliano et al., 2010; Mcgill et al., 2002; Wellbrock et al., 2008) (Figure 3). Thereby, MITF can favour survival by regulating genes such as *BCL2, BIRC7* and *BCL2A1* (Haq et al., 2013b; Mcgill et al., 2002), which has led to its description as lineage‐specific ‘survival oncogene’ (Garraway et al., 2005).

## The complex role of MITF in balancing proliferation and differentiation

Despite MITF's essential function in melanoma cell proliferation, it also consistently regulates the expression of differentiation genes (Figure 3). It is generally thought that high MITF expression levels are required to trigger differentiation after cell cycle exit (Busca and Ballotti, 2000). However, MITF^high^ cells are described as having the highest proliferative activity (Hoek et al., 2006), and in certain zebrafish mutants, melanocyte differentiation occurs also at low levels of functional MITF (albeit at a reduced rate (Zeng et al., 2015)). Moreover, cell division of pigmented melanocytes is also observed in zebrafish, but the frequency is increased at low levels of functional MITF, suggesting it is easier for these cells to re‐enter the cell cycle (Taylor et al., 2011). These findings not only reveal a great plasticity with regard to the balance between differentiation and proliferation in pigment cells, they also suggest that this balance is not strictly regulated by the steady‐state levels of functional MITF. Thus, under proliferative conditions, additional cellular signalling must interact with MITF and possibly its targets to favour cell cycle progression over the initiation of melanogenesis. The MAPK pathway could provide such signalling (Gray‐Schopfer et al., 2007), and indeed, long‐term BRAF or MEK inhibitor treatment of melanoma cells can trigger melanogenesis (Haq et al., 2013a). On the other hand, factors present in the microenvironment can tip the proliferation favourable balance and induce cell cycle exit and differentiation. Such a situation might explain why pigmented cells are frequently found in melanomas independent of the stage of disease.

The major signal triggering melanogenesis in melanocytes is based on cAMP‐induced CREB activation, which leads to a profound increase in the expression of MITF and its differentiation target genes, cell cycle exit and initiation of melanogenesis. Although MITF has been linked to the antiproliferative activity of cAMP signalling, the main effector of this response remains to be identified, because the two potential candidates p16INK4A and p21CIP1 appear not to be involved (Wellbrock and Marais, 2005). Whereas MITF induces cell cycle exit through p16INK4A in melanocytes (Loercher et al., 2005), cAMP inhibits melanoma cell growth in the absence of p16INK4A. Furthermore, although exogenous MITF causes a *p21CIP*1‐dependent cell cycle arrest in mouse fibroblasts and is a bona fide MITF target gene in melanoma cells (Carreira et al., 2005), cAMP – despite upregulating MITF – does not provoke an increase in p21CIP1 expression in melanoma cells (Wellbrock and Marais, 2005). Thus, in melanoma cells, p21CIP1 as MITF target might play another role than inhibiting the cell cycle. In line with this, SOX9 inhibits melanoma cell growth and induces the expression of MITF and p21CIP1, but the p21CIP1 growth inhibitory effect is largely independent of MITF (Passeron et al., 2009). Together, the expression of a MITF‐induced ‘antiproliferative’ factor in the context of cAMP signalling might depend on the concomitant activation of additional signals, a scenario that would reconcile high MITF expression levels with melanoma cell proliferation.

## MITF impacts on invasion

While MITF's function in survival, proliferation and differentiation is well understood, findings regarding its role in invasion are somewhat inconsistent (Figure 3). For instance, ectopic overexpression of MITF in 501mel cells enhances the invasive capacity of these cells in response to HGF (Mcgill et al., 2006). This pro‐invasive role of MITF is also seen in other cell lines, because the HGF receptor c‐MET is an MITF target gene (Beuret et al., 2007; Mcgill et al., 2006). Furthermore, the ‘melanoma predisposition’ mutant MITF^E316K^, which displays altered sumoylation, also significantly enhances 501mel invasion (Bertolotto et al., 2011). Strikingly however, increased invasion of 501mel cells can also be achieved by MITF depletion (Arozarena et al., 2011b; Carreira et al., 2006; Javelaud et al., 2011). An answer for this discrepancy might lie in the fact that 501mel cells express mutant hyperactive *β*‐catenin, and in these cells, MITF interferes with *β*‐catenin‐mediated transcription (Arozarena et al., 2011b). Hence, MITF depletion reveals the pro‐invasive function of *β*‐catenin.

Nevertheless, whereas manipulation of MITF expression might lead to confusing results regarding invasion, a role for MITF as suppressor of invasion is supported by the observation that several factors that reduce MITF expression, for example WNT5 or hypoxia (Cheli et al., 2012; Dissanayake et al., 2008; Feige et al., 2011), also increase invasiveness (Weeraratna et al., 2002; Widmer et al., 2013). Furthermore, AXL^+^/WNT5^+^ melanoma cells, lacking MITF expression, are highly invasive (Muller et al., 2014; Sensi et al., 2011). However, the mechanism as to how MITF regulates invasion is not entirely clear.

MITF is thought to suppress RHO‐/ROCK‐mediated invasion through *DIA1*, a target gene of MITF (Carreira et al., 2006). Indeed, as an actin nucleator, DIA1 regulates ROCK‐mediated changes in the actin cytoskeleton in a 2D setting (Carreira et al., 2006). However, in a 3D matrix environment, where RHO/ROCK signalling is relevant for invasion, DIA1 is dispensable and does not phenocopy MITF‐induced effects on ROCK and the actin cytoskeleton (Arozarena et al., 2011a). Thus, it appears that the effectors responsible for suppressing invasion downstream of MITF remain to be identified.

Because of the inverse expression of BRN2 and MITF, *in vivo* it was assumed that BRN2 regulates invasion by suppressing MITF expression. This theory was tested in an elegant *in vivo* study, in which a BRN2 reporter (GFP) and cellular pigmentation (as surrogate marker for MITF expression) were analysed during B16F2 melanoma progression (Pinner et al., 2009). As predicted, motile cells generally contained low levels of pigment. However, there was no simple inverse correlation of the BRN2 signal with levels of pigmentation (Pinner et al., 2009). This indicated that BRN2 expression levels were not correlated with levels of MITF. Thus, either BRN2 did not suppress MITF expression or MITF expression was not linked to pigmentation. Unfortunately, MITF expression levels were not analysed in this study, but the findings indicated that increased BRN2 expression favours motility. Indeed, BRN2 can induce melanoma cell invasion by suppressing *PDE5* (Arozarena et al., 2011b), and a prospective cohort study has linked the use of the PDE5 inhibitor sildenafil with an increased risk of melanoma (Li et al., 2014). While this emphasizes the relevance of BRN2 function for melanoma, the role of MITF was not assessed in this study.

## MITF regulates the tumourigenic and metastatic potential of melanoma

Several studies have shown that reduction in MITF expression reduces xenograft growth (Feige et al., 2011; Nakai et al., 2007), but others found that it increases tumour volume (Cheli et al., 2011, 2012) (Figure 3). These observed differences might be due to the timing and duration of MITF depletion. Pretreatment leading to the generation of cells with suppressed MITF expression appears to enhance tumour initiation, when eventually MITF expression can recover (Cheli et al., 2011, 2012), while continuous suppression of MITF is incompatible with tumour growth (Feige et al., 2011; Nakai et al., 2007). In line with this, melanoma cell lines that lack MITF expression grow tumours less frequently and tumour onset is delayed by several months compared to MITF‐expressing cell lines (Hoek et al., 2008). Furthermore, the ablation of functional MITF expression in BRAF^V600E^ induced melanomas in zebrafish leads to tumour regression (Lister et al., 2014). While this clearly indicates that MITF expression is required for the maintenance of melanoma growth, transient MITF depletion enhances the metastatic potential of melanoma cells in a tail vein injection assay (Cheli et al., 2012). This might be due to MITF acting as a suppressor of genes that are linked to enhanced metastasis seeding and implies an antimetastatic function for MITF. However, in a heterogeneous tumour, the situation is more complex and MITF's role in metastasis awaits further elucidation.

For some time now, the ‘MITF rheostat’ model (Carreira et al., 2006; Hoek and Goding, 2010) has linked levels of MITF ‘activity’ to distinct melanoma cell behaviours and phenotypes. While the term ‘activity’ has never been accurately defined, the majority of properties assigned to MITF ‘activity’ are in fact linked to its ‘expression levels’, simply because they are based on depletion or overexpression experiments. Examples are the stimulation of differentiation after cAMP‐induced upregulation of MITF expression (high activity) or G1 arrested cells with an invasive, ‘stem cell‐like’ phenotype after RNAi‐mediated MITF depletion (low activity). Using these criteria, the rheostat model suggests that melanoma cells – depending on their MITF activity level – are either proliferating or invading (Carreira et al., 2006). However, a recent elegant study applying real‐time cell cycle imaging *in vitro* in 3D and *in vivo* revealed that these activities are not mutually exclusive (Haass et al., 2014). Moreover, it is increasingly apparent that MITF also does opposing things at similar expression levels when in a different context (see Figure 3), such as inhibiting *β*‐catenin‐ or stimulating HGF‐mediated invasion. Thus, steady‐state MITF levels alone are not informative enough.

Undoubtedly, the abundance of MITF will influence its occupancy at target gene promoters. However, different signalling conditions will post‐translationally modify MITF and impact on its target gene repertoire by regulating DNA binding specificity/affinity and the interaction with co‐factors. Thus, there will not be only ‘high’ or ‘low’ MITF activity, but ‘differential’ activity, and assigning MITF only one particular function or phenotype at a certain level, as it is carried out in the rheostat model (Carreira et al., 2006) might be too restrictive, and we have to consider new ways of describing MITF function in melanoma.

## MITF expression plasticity and tumour heterogeneity

The majority of melanomas display great heterogeneity not only with regard to MITF‐negative and MITF‐positive populations, but also with regard to expression levels amongst the MITF‐positive cells. This was clearly revealed in a recent study assessing ‘MITF‐related’ gene expression signatures in single cells (Ennen et al., 2014). These gene expression signatures called ‘proliferative’ and ‘invasive’ were originally defined by Hoek and co‐workers (Hoek et al., 2006). They are closely linked to MITF expression and describe the MITF‐positive and the MITF‐negative (AXL^+^/WNT5A^+^) population, respectively. As such, it is expected that MITF‐positive cells display a ‘proliferative’ gene signature, whereas MITF‐negative cells exhibit an ‘invasive signature’.

Employing these signatures, Ennen and co‐workers found that within a single MITF‐positive cell line, individual cells displayed significant heterogeneity in MITF expression levels and gene signatures. Moreover, some cells despite expressing high levels of MITF represented with an ‘invasive’ signature (Ennen et al., 2014). Importantly, this heterogeneity was maintained *in vivo*. In contrast, however, a AXL^+^/WNT5A^+^ cell line that expressed no detectable levels of MITF displayed a low level of heterogeneity with regard to the signatures, probably reflecting that these MITF‐negative cells are not governed by MITF and that the ‘invasive’ signature in these cells is regulated by another factor. Furthermore, tumours from MITF‐negative cells stayed negative and did not ‘re‐express’ MITF to significant levels (Ennen et al., 2014).

This study has revealed some important findings that should be considered with regard to current models describing melanoma plasticity and heterogeneity. For instance, the MITF‐centred EMT‐like concept of ‘phenotype switching’ proposes that tumour progression and heterogeneity are driven by dynamic reversible transitions between cell states, which are defined by the ‘proliferative’ and ‘invasive’ gene expression signatures (Hoek et al., 2006, 2008). This model is based on the idea that cells switch back and forth between different MITF expression and ‘activity’ states. Applying this model to the MITF‐positive and MITF‐negative populations would predict that melanoma cells reversibly switch between AXL^‐^/WNT5A^‐^ and AXL^+^/WNT5A^+^ cell populations.

However, while Ennen et al. found an intrinsic dynamic with regard to MITF expression and ‘AXL^+^/WNT5A^+^ ‐related’ signatures in the MITF‐positive cells, suggesting a partial phenotype switch, they did not detect that MITF‐negative (AXL^+^/WNT5A^+^) cells switch their signature, even *in vivo*, where these cells were exposed to a plethora of exogenous factors that could induce MITF expression.

Dynamic switching in phenotypes clearly occurs in MITF‐positive cells, which appear to be predisposed to exogenous signals that can induce a change in cellular behaviour. For instance, a reversible switch between populations of undifferentiated and differentiated MITF‐positive cells can be observed during melanoma progression (Pinner et al., 2009). However, this shift does not depend on a change in MITF expression level and can be explained by the presence of microenvironment‐derived factors, which could induce a cell cycle arrest and enable MITF (and other proteins) to trigger differentiation. The absence of such a factor would then allow cells to re‐enter the cell cycle and de‐differentiate over time. Interestingly, a similar situation is found with MAPK pathway inhibitors, which arrest the cell cycle and trigger melanogenesis (Haq et al., 2013a); again, the removal of drug allows a reversion of this switch. Alternatively, exogenous factors such as WNT5A or hypoxia can reduce MITF expression in MITF‐positive cells and produce a phenotype with increased invasiveness and metastatic potential (Cheli et al., 2012; O'connell et al., 2013; Widmer et al., 2013). Nevertheless, it is not clear that these factors induce an epigenetic change and turn the cells permanently into AXL^+^/WNT5A^+^ MITF‐negative cells. It is possible, however, that such cells are generated by other mechanisms. *MITF* hypermethylation can be detected in melanomas and in melanoma cell lines, and moreover, in these cells, MITF‐regulated differentiation genes are also hypermethylated (Lauss et al., 2015). Hence, possibly as the result of the accumulation of methylation events over time, individual melanoma cells become in fact locked in a relatively stable ‘MITF/differentiation‐off’ state. Indeed, some melanoma cell lines display a ‘neural precursor signature’, which intriguingly contains WNT5A and AXL (Tap et al., 2010).

In summary, MITF‐positive cells display a reversible dynamic with regard to MITF expression and function, which can be triggered by the microenvironment. However, a switch from MITF‐negative to MITF‐positive cells possibly represents a rare event that requires the manifestation of epigenetic alterations, such as demethylation (Lauss et al., 2015). In this context, it is striking that ~14% of melanomas stain positive for MITF and AXL, but it is not clear, whether in these tumours, cells have undergone partial switching. Nevertheless, this indicates that a third ‘class’ of melanoma cells exists where a particular intracellular signalling permits the co‐existence of MITF with AXL and WNT5A, which otherwise is mutual exclusive (Dissanayake et al., 2008; Konieczkowski et al., 2014).

If epigenetic switching with regard to MITF expression is a rare event during melanoma progression, additional processes might contribute to the maintenance of heterogeneity with regard to MITF‐positive and MITF‐negative populations. One such process could be ‘co‐operativity’, whereby cancer cells with different ‘phenotypes’ communicate and collectively perform an activity required for efficient progression. *In vivo* single‐cell analysis has revealed that non‐invasive melanoma cells can co‐operate with invasive cells and thereby ‘piggyback’ along with invasive cells, thus maintaining heterogeneity while disseminating (Chapman et al., 2014). Co‐operativity also agrees with the heterogeneity of MITF expression detected in circulating melanoma cell clusters (Khoja et al., 2014). Interestingly, an analogous heterogeneity amongst circulating tumour cells also exists in the context of EMT (Hou et al., 2011; Yu et al., 2013), and comparable co‐operative behaviour between EMT and non‐EMT cells has been described. Thus, while switching of cell activities occurs frequently amongst MITF‐positive cells and can generate cells with proliferative and invasive capacities, co‐operativity can enable the maintenance of various phenotypes without selective pressure.

Apart from its effect on melanoma progression, MITF heterogeneity also will impact on MAPK pathway targeted therapy. In high MITF‐expressing cells, treatment is hindered through increased tolerance, but might benefit from enhanced antigen presentation. On the other hand, subpopulations of AXL^+^/WNT5A^+^ MITF‐negative cells can escape therapy and thus will be able to re‐establish tumour growth. The complexity of this situation is exclusively revealed in a patient, who relapsed on vemurafenib with both MITF‐positive and MITF‐negative resistant clones (Muller et al., 2014). This clearly highlights that in future, we not only need to improve our understanding of MITF function in MITF‐positive melanoma cells and identify which factors govern MITF‐negative cells, but we also need to comprehend how MITF‐positive cells interact with MITF‐negative cells in the context of melanoma progression and therapy.
